# Brain-Derived Neurotrophic Factor Elevates Activating Transcription Factor 4 (ATF4) in Neurons and Promotes ATF4-Dependent Induction of *Sesn2*

**DOI:** 10.3389/fnmol.2018.00062

**Published:** 2018-03-01

**Authors:** Jin Liu, Fatou Amar, Carlo Corona, Raphaella W. L. So, Stuart J. Andrews, Peter L. Nagy, Michael L. Shelanski, Lloyd A. Greene

**Affiliations:** Department of Pathology and Cell Biology, Columbia University College of Physicians and Surgeons, New York, NY, United States

**Keywords:** brain-derived neurotrophic factor (BDNF), transcription factor, neuron, gene regulation, neurotrophin, activating transcription factor 4 (ATF4), Sestrin2

## Abstract

Activating transcription factor 4 (ATF4) plays important physiologic roles in the brain including regulation of learning and memory as well as neuronal survival and death. Yet, outside of translational regulation by the eIF2α-dependent stress response pathway, there is little information about how its levels are controlled in neurons. Here, we show that brain-derived neurotrophic factor (BDNF) promotes a rapid and sustained increase in neuronal ATF4 transcripts and protein levels. This increase is dependent on tropomyosin receptor kinase (TrkB) signaling, but independent of levels of phosphorylated eIF2α. The elevation in ATF4 protein occurs both in nuclei and processes. Transcriptome analysis revealed that ATF4 mediates BDNF-promoted induction of *Sesn2* which encodes Sestrin2, a protector against oxidative and genotoxic stresses and a mTor complex 1 inhibitor. In contrast, BDNF-elevated ATF4 did not affect expression of a number of other known ATF4 targets including several with pro-apoptotic activity. The capacity of BDNF to elevate neuronal ATF4 may thus represent a means to maintain this transcription factor at levels that provide neuroprotection and optimal brain function without risk of triggering neurodegeneration.

## Introduction

Activating transcription factor 4 (ATF4; also referred to as CREB2) is a ubiquitously expressed member of the activating transcription factor/CAMP Responsive Element Binding Protein (CREB) family that plays multiple roles in cellular physiology and pathology (Rutkowski and Kaufman, 2003; Ameri and Harris, 2008). Among the most studied of these is its role as a responder to stresses such as accumulation of unfolded proteins and amino acid deprivation (Rutkowski and Kaufman, 2003; Ameri and Harris, 2008; Kilberg et al., 2009; Pakos-Zebrucka et al., 2016). Cellular levels of ATF4 rapidly increase under such conditions and these in turn regulate transcription of a variety of genes that can lead either to alleviation of the stress or to cell death (Lange et al., 2008; Sun et al., 2013; Baleriola et al., 2014; Iurlaro and Muñoz-Pinedo, 2016; Pakos-Zebrucka et al., 2016).

ATF4 appears to be universally expressed including in the nervous system. Within neurons, ATF4 is present not only in nuclei, but also in processes and synapses and there is evidence that it is retrogradely transported to the nucleus, thus suggesting that it can function to respond to local events and then affect transcription (Lai et al., 2008; Sun et al., 2013; Baleriola et al., 2014). Studies to date have uncovered several seemingly diverse roles for ATF4 in the brain. In response to pathological stresses, ATF4 has been described as either promoting or suppressing neuron dysfunction or death (Green et al., 2008; Lange et al., 2008; Sun et al., 2013; Baleriola et al., 2014; Rittiner et al., 2016). Additional studies have characterized ATF4 as a controller of neurogenesis (Frank et al., 2010) and a component of a neuronal circadian rhythm pathway (Ushijima et al., 2012). ATF4 has also been portrayed as an important modulator of neuronal plasticity and various lines of evidence have implicated it as either a positive or a negative regulator of plasticity and memory (Costa-Mattioli et al., 2007; Kandel, 2012; Trinh et al., 2012; Liu et al., 2014; Hu et al., 2015; Pasini et al., 2015). Studies from our lab have shown that experimental depletion of neuronal ATF4 impairs spatial memory as well as multiple aspects of synaptic function, suggesting the importance of maintaining appropriate levels of ATF4 in neurons (Liu et al., 2014; Pasini et al., 2015).

Given ATF4’s many activities, it is important to understand how its levels are regulated. The best characterized mechanism of ATF4 regulation is via phosphorylation of the eukaryotic translation initiation factor eIF2α (Kilberg et al., 2009; Bellato and Hajj, 2016; Iurlaro and Muñoz-Pinedo, 2016; Pakos-Zebrucka et al., 2016). A variety of cell stresses promote activation of protein kinases that phosphorylate eIF2α leading to selectively elevated *Atf4* translation. It also has been suggested that eIF2α phosphorylation acts as a switch to modulate ATF4 expression in the context of its functions in neuronal plasticity (Costa-Mattioli et al., 2007). In addition to translational regulation, ATF4 levels are also subject to modulation by transcriptional induction (Dey et al., 2010) and by degradation (Ameri et al., 2004).

In addition to cellular stresses, there are several instances in which growth factors have been reported to upregulate ATF4 (Malabanan et al., 2008, 2012). The neurotrophin, brain-derived neurotrophic factor (BDNF) is among the major growth factors present in the nervous system. As such, BDNF affects a wide array of properties in neurons including differentiation, connectivity, survival, metabolism excitability, repair and regeneration (Gottmann et al., 2009; Numakawa et al., 2010; Yoshii and Constantine-Paton, 2010; Lu et al., 2014; Marosi and Mattson, 2014; Zagrebelsky and Korte, 2014; Gonzalez et al., 2016). At the organismal level this translates into regulation of learning and memory and of a range of behaviors. A variety of normal non-neuronal cells and tumor cell types also display responses to BDNF (Thiele et al., 2009; Marosi and Mattson, 2014; Chopin et al., 2016). Many of the proximal signaling events that follow binding of BDNF to the tropomyosin receptor kinase (TrkB) and to the p75 nerve growth factor receptor have been described in detail as have been consequent downstream transcription-dependent and -independent responses (Reichardt, 2006; Gottmann et al., 2009; Numakawa et al., 2010; Yoshii and Constantine-Paton, 2010; Gonzalez et al., 2016). However, the transcription factors that are regulated by and that mediate the genomic responses to BDNF and that underlie many of its actions have been less extensively characterized. In light of these issues, we therefore explored the possibility that BDNF might regulate neuronal ATF4 expression.

Here, we report that BDNF promotes a rapid and sustained elevation of ATF4 mRNA and protein levels in cortical and hippocampal neurons via activation of TrkB receptors. The increase in ATF4 protein occurs in processes as well as in cell bodies and nuclei. We also show that ATF4 mediates BDNF-promoted induction of the neuroprotective gene *Sesn2*. These findings identify ATF4 as an element of the BDNF signaling mechanism and suggest that BDNF may contribute to maintenance of optimal ATF4 levels in neurons.

## Materials and Methods

### DNA Constructs and Lentivirus Preparation

All lentiviral constructs were produced as previously described (Liu et al., 2014). Lentiviral particles were generated by using the 2nd generation packaging system from Addgene (Liu et al., 2014). Briefly, lentiviral constructs for shRNA or overexpression were co-transfected with the packaging vectors into HEK293T cells by the calcium phosphate method. Supernatants containing virus were collected 24 h and 48 h after transfection. The viruses were concentrated 20–30× by centrifugation in an Amicon Ultra centrifugal filter (100K; Millipore) following the manufacturer’s instructions. Viruses were aliquoted and stored at −80°C. Viral titers ranged 1–5 × 10^6^ infectious units/μl.

### Cell Culture

HEK293T cells were purchased from the ATCC and maintained in DMEM medium (Invitrogen) containing 10% FBS (Hyclone). For virus preparation, the calcium phosphate method was used for DNA transfection.

Primary hippocampal and cortical cultures were prepared as previously described (Liu et al., 2014). Briefly, hippocampi or cortices from E18 rat embryo brains were collected, treated with trypsin, and plated on poly-D-lysine (Sigma) coated plates or coverslips in 12-well plates at a density of 3 × 10^5^/well. For cell staining experiments, neurons were cultured at a low density (3 × 10^4^/well) on cover glasses and maintained in conditioned medium (from regular density cultures). Neurons were maintained in Neurobasal medium (Invitrogen) supplemented with 2% B-27 (Invitrogen) and 0.5 mM glutamine (Invitrogen). Half of the culture medium was changed every 3 days after plating. All animal use was performed according to protocols examined and approved by the Animal Use and Care Committee of Columbia University.

### BDNF Treatment

For most experiments, BDNF (Sigma) was added to cultured neurons at 12 DIV (Days *in vitro*) for 4 h. Cells were collected at indicated time points. In most cases, the concentration of BDNF was 25 ng/ml, unless indicated in the text or figures. For long-term (more than 1 day) treatment, BDNF was added every day at the same concentration. For the K252a experiments, 0.2 μM K252a (Sigma) was added to the cultured neurons 1 h before BDNF treatment and was maintained in the medium during treatment. For TrkB Fc-IgG experiments, 1 μg/ml TrkB Fc-IgG and control IgG (R&D Systems) were added to the cultured neurons 1 h before BDNF treatment and was retained in the medium during treatment. Acute and gradual BDNF treatment was as previously described (Ji et al., 2010). For actinomycin-D experiments, 0.2 μM actinomycin D (Sigma) was added to the cultured neurons 0.5 h before BDNF treatment and was retained in the medium with BDNF for 2 h. For experiments with ISRIB (Integrated Stress Response inhibitor, trans-N,N′-(Cyclohexane-1,4-diyl)bis(2-(4-chlorophenoxy)acetamide, trans-N,N′-1,4-Cyclohexanediylbis[2-(4-chlorophenoxy)-acetamide), cultures were incubated with 50 nM ISRIB (Sigma) for 1 h prior to addition of BDNF.

### Western Immunoblotting

For Western immunoblotting analysis, infected neurons were collected in 1× LDS loading buffer (Invitrogen) containing 2.5% β-mercaptoethanol and boiled for 10 min. Proteins were separated by electrophoresis in 10% NuPAGE gels and immunoblotting was carried out as previously described (Liu et al., 2014). Densitometric analysis of the bands was performed using the ImageJ program. Comparison of data and calculation of *p* values were performed by using Student’s *t*-test.

The following primary antibodies were used: rabbit anti-ATF4 (1:1000, Cell Signaling), anti-phospho-eIF2α (Ser51; 1:1000, Cell Signaling) and mouse anti-GAPDH (1:2000; Imgenex). The anti-ATF4 has been validated by loss of signal after ATF4 knockdown with shRNAs (Liu et al., 2014; Pasini et al., 2015).

### Real-Time Quantitative PCR, Chip Analysis and RNAseq

To assess the *Atf4* message levels in cultured neurons, total RNA was isolated at indicated time points after BDNF treatment by using RNeasy Mini Kit (Qiagen). Reverse transcription was performed by using the First-strand cDNA Synthesis Kit (Origene) following the manufacturer’s instructions. Quantitative PCR was carried out in a “realplex2” machine (Eppendorf) by using the following primer pair for ATF4: 5′-GC TTGTCGGGACCCAAATTG-3′ and 5′-ACACCTGCGGCTCT TCTTCG-3′. The primers for Sesn-2 were: 5′-TTGCCTGCTA CCCGGAGAAG-3′ and 5′-TGGCGCGGAGGGCATAGAG-3′. PCR with a primer for α-tubulin was used to normalize RNA levels.

For RNAseq, lenti-shATF4 and lenti-shCtrl viruses were used to infect cultured hippocampal neurons on 7 DIV; cells were treated with BDNF on 12 DIV; RNA samples were collected at indicated time points with three cultures per condition. The infection efficiency was determined to be 91.7% (*n* = 36). The coding RNA library was generated using the TruSeq Stranded Total RNA LT Sample Prep kit (Illumina) which includes rRNA depletion and chemical fragmentation. RNA was sequenced with 125 cycles × 2 paired-end sequencing on the HiSeq2500 following the manufacturer’s recommendations. For transcriptome analysis, fastq files from CASAVA were filtered for ribosomal RNA (rRNA) using SortMeRNA (v.1.7)^1^ and trimmed to remove poor-quality tails using TrimGalore (v.0.2.7)^2^ with settings to exclude reads of quality score <20 and read length <20. Of the 24 ribosomal-RNA depleted samples, we obtained 55–113 m reads (mean 85 m). Only 0.9%–3.5% (mean 1.4%) of the reads were removed by the SortMeRNA software. Remaining reads were mapped to the transcriptome of the Rattus norvegicus genome (Rnor_5.0/release_79) provided by Ensembl (Kersey et al., 2016) which is based on NCBI GenBank assembly GCA_000001895.3 (Benson et al., 2006) provided by the Rat Genome Sequencing Consortium (Gibbs et al., 2004). Mapping was performed using the Tuxedo Suite (Trapnell et al., 2012, 2014) consisting of TopHat2 (v.2.0.8), BOWTIE2 (v.2.1.0), and CUFFLINKS (v.2.1.1). Non-uniquely mapped reads were excluded before estimation of fragments per kilobase per million reads (FPKMs) by CUFFLINKS. At least 50 million uniquely mapped reads were required per sample with less than 5% DNA contamination. The data have been deposited in ArrayExpress with accession number E-MTAB-6046.

For GeneChip microarray analysis, cultured cortical neurons were infected with shATF4, ATF4 and control lentiviruses at 5 DIV and RNA samples were collected at 8 DIV. Three independent samples of each type were subjected to analysis using the rat genome U34 set (Affymetrix) according to the manufacturer’s instructions.

### Analysis of RNAseq Data Using “FPKM Time Course Explorer”

An interactive web-based application, “FPKM Time Course Explorer”, was developed using the “shiny” package (Hornik, 2016; Chang et al., 2017) to help with the visualization and statistical analysis of the gene-expression time-series data.

The application visualizes time-series profiles defined by the commonly-used “fragments per kilobase per megabase” (FPKM) measure of gene expression. FPKM was defined as COUNT_gene/(LENGTH_gene * DEPTH), where COUNT_gene is the number of reads mapping to the transcript for the gene, LENGTH_gene is the length of the transcript for the gene, and DEPTH is the total number of reads mapping to the transcriptome overall. Gene expression profiles for a given sample-gene pair were obtained by listing chronologically the FPKM values of the basal state and various times (2, 4, 8, 16 and 24 h) after treatment with BDNF. Profiles were displayed in pairs to allow direct comparison of the knockdown and control samples.

The application allows the user to filter and rank the profiles according to a number of summary statistics for each treated/untreated pair. The most basic filter included and/or excluded profiles corresponding to sets of genes chosen by the user. The second filter excluded profiles whose mean FPKM was below a noise threshold value set by the user. The third filter excluded profiles whose ranking score (defined below) fell outside of an interval (min_rank, max_rank) chosen by the user. By changing the noise threshold, ranking scores and ranking interval, as well as the sets of included and excluded genes, the user was able to explore the five-dimensional space of gene-expression profiles to draw meaningful biological insights from this large time-series dataset.

### Confocal Imaging

Mature hippocampal neurons were treated with BDNF and fixed with 4% paraformaldehyde (PFA) after 15 mins or 4 h for 10 min at room temperature. Neurons were washed with PBS, permeabilized with PBS+0.25% Triton-X100 and blocked for 1 h with 1% BSA in PBS+0.25% Triton-X100. Primary antibody was incubated overnight at 4°C. Triple label immunofluorescence was performed using Alexa Fluor 488, 555-conjugated secondary antibodies (1:500, Molecular Probes, Invitrogen) and Hoescht 33342 for nuclear staining. Digital images were obtained using a Zeiss (LSM800) microscope. Raw image z-stacks (0.4 μm intervals) were analyzed using ImageJ (nih.gov).

The following primary antibodies were used: rabbit anti-ATF4 (1:300; Liu et al., 2014) and anti-beta III Tubulin (1:300, Novus).

Quantification of ATF4 levels in somas and processes was carried out as follows. ATF4 levels in somas were quantified by measuring green (ATF4) and red (tubulin) fluorescence intensity in the cell body using ImageJ, and then normalizing the green to the red signal. To measure ATF4 levels in the processes, green (ATF4) and red (tubulin) fluorescence intensities were measured in the entire field using ImageJ followed by normalization of the green to the red signal. Then the normalized ATF4 signal from all the cell bodies in the field was subtracted from the normalized total signal. In each case, the normalized values for ATF4 intensity are reported as arbitrary units (A.U.).

### Fractionation

For the generation of nuclear- and synaptosomal-enriched protein fractions, DIV 21 hippocampal neurons were placed on ice immediately following treatment and washed with cold PBS. Material from six wells of a 12-well plate were pooled together as an n of 1. The six wells were scraped sequentially with 250 μl of synaptosome buffer (1 mM MgCl_2_ and 0.32 M sucrose with Halt Protease and Phosphatase Inhibitor Cocktail (Thermo Scientific #78442, 100×) targeting aminopeptidases, cysteine and serine proteases and serine/threonine and protein tyrosine phosphatases and then the final pools of lysates were transferred into 1.5 ml Eppendorf tubes. Cell lysates were homogenized with a Teflon homogenizer (15 strokes). Homogenates were centrifuged at 2100 rpm (470 *g*) for 2 min at 4°C. Supernatant was removed and placed in a new tube. The remaining pellet was designated the P1 fraction (nuclear enriched). The supernatant was then centrifuged at 9700 rpm (10,000 *g*) for 10 min. The resulting pellet was designated the P2 (synaptosomal enriched). Each pellet was resuspended in 100 μl of 1× LDS loading buffer (Invitrogen), vortexed for 1 min and boiled for 5 min followed by western immunoblotting analysis.

### Statistical Analysis of Data

Statistical analysis of data for individual experiments was carried out as described in legends. For comparison of mean values for two groups, unpaired *t*-tests were carried out using Graphpad. For experiments involving multiple measurements compared with a control value (time courses or dose-responses) we used the Kruskal-Wallis one-way analysis of variance test^3^. In the cases in which the test yielded a significant *p* value (<0.05), *post hoc* analysis was carried out to identify pairs of samples with significant differences using the Conover method adjusted by the false-discovery rate (FDR) procedure of Benjamini-Hochberg. For experiments in which multiple treatments were compared with a control condition, we used one-way ANOVA with the *post hoc* Tukey HSD test^4^.

## Results

### BDNF Elevates ATF4 Protein Expression in Cultured Cortical and Hippocampal Neurons

**Figure 1:**
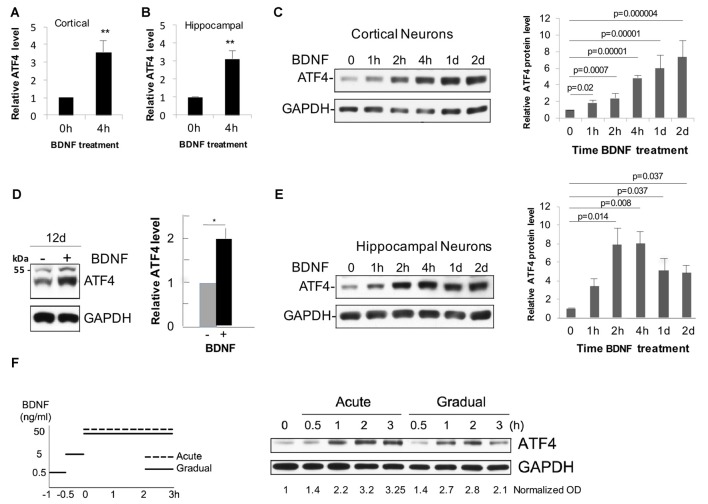
Brain-derived neurotrophic factor (BDNF) promotes rapid and sustained elevation of neuronal activating transcription factor 4 (ATF4) protein expression in primary cultures of rat cortical and hippocampal neurons. **(A)** Mean elevation of ATF4 protein levels in response to BDNF treatment (4 h, 25 ng/ml) determined by Western immunoblotting in cultures of rat cortical neurons (12 Days *in vitro* (DIV)). Values (normalized to GAPDH signal for loading control here and in subsequent panels and figures) are relative to levels in non-treated control cultures and represent mean ± SEM from 10 independent experiments, each performed with lysates from 1 to 3 replicate (treated and control) cultures. **p = 0.0016 (*t*-test). **(B)** Mean elevation of ATF4 protein levels in response to BDNF treatment (4 h, 25 ng/ml) determined by Western immunoblotting in cultures of rat hippocampal neurons (12 DIV). Values are normalized to levels in non-treated control cultures and represent mean ± SEM from 20 independent experiments, each performed with lysates from 1 to 3 replicate (treated and control) cultures. **p = 0.0006 (*t*-test). **(C)** Time course of BDNF-stimulated (25 ng/ml) increases in ATF4 protein in cultured (12 DIV) cortical neurons as determined by Western immunoblotting. Left-hand panel shows representative immunoblot, right-hand panel shows quantification from 3 to 4 independent experiments, each with one sample per time point. Values are normalized to levels in non-treated control cultures and represent mean ± SEM. *p* values were calculated vs. 0 time using the Kruskal-Wallis test (overall *p* = 0.003) with the *post hoc* Conover method adjusted by the false-discovery rate (FDR) procedure of Benjamini-Hochberg. **(D)** BDNF sustains elevation of ATF4 protein levels in cultured cortical neurons. Cultures were maintained for 10 days without BDNF and then for an additional 12 days ± BDNF (25 ng/ml) and then harvested for ATF4 expression by Western immunoblotting. Left panel shows representative immunoblot, right panel quantification from three independent experiments, each with one sample per time point. *p value (*p* = 0.013) calculated by *t*-test. The band at the 55 kDa marker on the blot is non-specific (Liu et al., 2014). **(E)** Time course of BDNF-stimulated (25 ng/ml) increases in ATF4 protein in cultured (12 DIV) hippocampal neurons as determined by Western immunoblotting. Left-hand panel shows representative immunoblot, right-hand panel shows quantification from 3 to 4 independent experiments, each with one sample per time point. Values are normalized to levels in non-treated control cultures and represent mean ± SEM. *p* values were calculated vs. 0 time using the Kruskal-Wallis test (overall *p* = 0.028) with the *post hoc* Conover method adjusted by the FDR procedure of Benjamini-Hochberg. **(F)** BDNF elevates ATF4 protein in cultured hippocampal neurons (12 DIV) for both acute and gradual treatment protocols. Left panel illustrates treatment protocols (Ji et al., 2010), right panel shows Western immunoblot of response. Normalized (to GAPDH) optical density values are given below the blot. Data represent a single experiment. Comparable results were obtained in an independent experiment with cortical neurons.

To assess whether BDNF affects neuronal ATF4 protein levels, we established cultures (12 DIV unless otherwise indicated) of rat cortical and hippocampal neurons. Both neuron types have been described as BDNF responsive (Knusel et al., 1992; Ip et al., 1993). Addition of 25–100 ng/ml of BDNF to the cultures elicited a consistent elevation of ATF4 protein expression as detected by Western immunoblotting with an antiserum previously verified to recognize rat ATF4 (Liu et al., 2014). While there was variation from experiment to experiment, over multiple independent experiments, the mean elevation of ATF4 protein levels after 4 h of BDNF treatment was about 3-fold in cortical cultures (*t*-test, *p* = 0.0016, *n* = 10 independent experiments) and 3.5-fold (*t*-test, *p* = 0.0006, *n* = 20 independent experiments) in hippocampal cultures (Figures 1A,B).

Time course studies in cortical cultures (Figures 1C,D) indicated that ATF4 expression was significantly increased compared to untreated cultures by 1 h of BDNF treatment, appeared to reach maximum elevation by about 4 h and remained significantly elevated at 1 and 2 days and at 12 days of treatment. Hippocampal cultures also showed significantly elevated ATF4 levels compared with untreated controls by 2 h of BDNF exposure and reached a maximum by 2–4 h of treatment with elevation still apparent at 1 and 2 days (Figure 1E).

It has been reported that acute vs. gradual addition of BDNF to cultures elicits distinctly different temporal patterns of signaling by 3–4 h of treatment (Ji et al., 2010). However, we did not observe striking differences in the time course of ATF4 regulation between the two protocols for hippocampal (Figure 1F) or cortical neurons (data not shown).

### BDNF Elevates ATF4 in Processes, Cell Bodies and Nuclei

**Figure 2:**
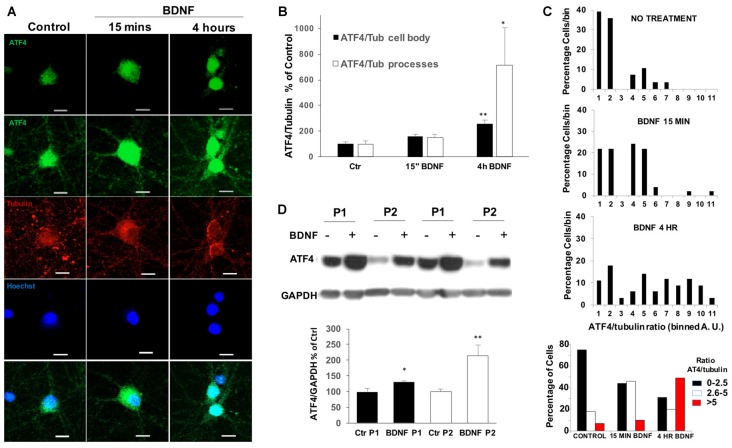
BDNF upregulates ATF4 expression in processes, cell bodies and nuclei of cultured hippocampal neurons. **(A)** DIV 21 hippocampal neurons were treated with 25 ng/ml of BDNF or vehicle for 15 mins or 4 h, then immunofluorescently labeled for ATF4 (green) and beta-3-tubulin (red) and for Hoescht 33342 staining (blue). The second row shows a longer exposure to reveal processes. Representative images are shown. Scale bar = 10 μm. **(B)** Quantification of the ATF4 immunostaining fluorescence intensity of cell bodies and processes for the cultured neurons described in **(A)**. Details for the separate quantification in cell bodies and processes are given in Experimental Procedures. Data were used to compute the intensity ratios for ATF4 vs. beta-3-tubulin at each time with the ratios at time 0 set to 100%. *n* = 28–42 randomly chosen cell bodies and 7–9 randomly chosen neurite fields per group. Values are means ± SEM. **p = 0.0001, *p = 0.008 compared with controls. *p* values were calculated vs. contol using the Kruskal-Wallis test (overall *p* = 0.002) with the *post hoc* Conover method adjusted by the FDR procedure of Benjamini-Hochberg. **(C)** BDNF changes the expression of ATF4 in a subpopulation of hippocampal neurons. The values (in arbitary units, A.U.) for ATF4/beta-3-tubulin immunofluorescence ratios in individual cell bodies were determined as in **(B)** and grouped into bins (from 1 A.U to 11 A.U. in the three upper panels and as indicated in the lower panel). Graphs show the distributions of percentages of cell bodies in each bin at different times of BDNF treatment. *n* = 28–42 randomly chosen cells per time point. **(D)** BDNF treatment (4 h, 25 ng/ml) increases ATF4 protein in both nuclear-enriched and synaptosomal-enriched cell fractions. DIV 21 hippocampal neurons treated with BDNF or vehicle were separated into nuclear-enriched (P1, black bars) and synaptosomal-enriched (P2, white bars) fractions as described in “Materials and Methods” section and ATF4 protein levels were determined by Western immunoblotting. Upper panel shows a representative immunoblot, lower panel quantification with values for control set at 100. Values are means ± SEM, *n* = 3 independent experiments, each with duplicate cell lysates. *p < 0.029, **p < 0.014 vs. controls (no BDNF), *t*-test.

We next used immunohistochemistry to assess the effect of BDNF on ATF4 protein levels in cultured hippocampal neurons. Before BDNF treatment, basal ATF4 expression was evident in all neurons (identified by co-staining with NeuN and beta-3-tubulin) in a highly punctate pattern within processes and cell bodies (Figure 2A). Increased immunostaining in a subset of neurons was apparent within 15 min of BDNF exposure and was even more apparent by 4 h in both neurites and cell bodies (Figure 2A). To quantify this effect, we measured the ratio of signals for both ATF4 and beta-3-tubulin in individual cell bodies as well as in processes. This revealed a significant increase of ATF4 in cell bodies of about 2.5-fold by 4 h (Figure 2B). For processes there was a significant increase in ATF4 staining of about 7-fold by 4 h of treatment. Because there appeared to be heterogeneity in the response, we grouped the individual values for cell body ATF4/beta-3-tubulin ratios into bins and plotted the results (Figure 2C). This indicated a shift to elevated ATF4 expression starting within 15 min and greatly increased by 4 h. The data also suggested that 30%–50% of the neurons did not show elevated cell body levels of ATF4 in response to BDNF. If this is the case, then the increases in ATF4 protein stimulated by BDNF per responsive neuron are up to 2-fold higher and more rapid than indicated in experiments using whole cultures.

Co-localization of ATF4 immunostaining with nuclear Hoescht 33342 staining indicated that nuclear levels of ATF4 were elevated by 4 h of BDNF treatment (Figure 2A). To examine this more closely, we prepared subcellular P1 (nuclear-enriched) and P2 (synaptosomal-enriched) fractions from cultured hippocampal neurons with or without 4 h of BDNF treatment. The quality of the nuclear fraction was verified by strong enrichment for NeuN (Supplementary Figure S1). Both the P1 and P2 fractions showed significant increases in ATF4 protein content after BDNF exposure (Figure 2D). The elevated levels of ATF4 in the nuclear fraction thus suggest that the induced ATF4 is appropriately positioned to affect gene regulation.

### Elevation of ATF4 Protein Levels by BDNF Requires TrkB Signaling

**Figure 3:**
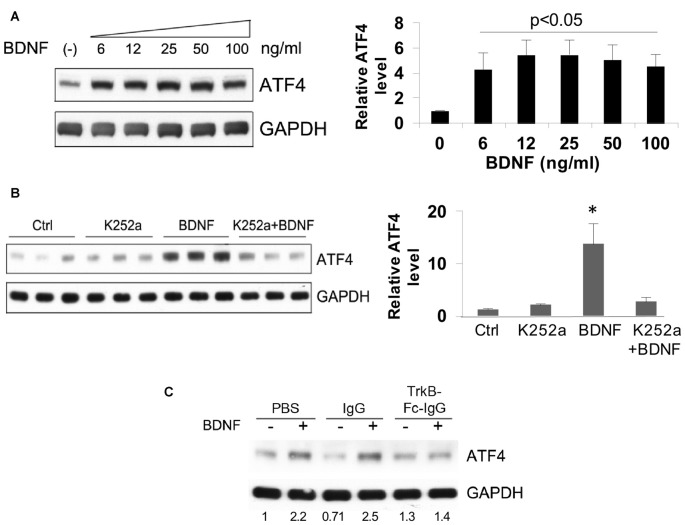
Elevation of ATF4 protein levels by BDNF requires tropomyosin receptor kinase (TrkB). **(A)** BDNF upregulates ATF4 protein expression in a dose-dependent manner. Cultured hippocampal neurons were treated with the indicated concentrations of BDNF for 4 h at 12 DIV. Left panel shows representative Western immunoblot, right panel quantification (means ± SEM for six independent experiments, each performed with a single lysate per concentration). *p* values were calculated vs. no BDNF treatment, using the Kruskal-Wallis test (overall *p* = 0.038) with the *post hoc* Conover method adjusted by the FDR procedure of Benjamini-Hochberg and were 0.042 (6 ng/ml), 0.02 (12 ng/ml), 0.02 (25 ng/ml), 0.035 (50 ng/ml) and 0.035 (100 ng/ml). The test revealed no significant differences (*p* = 0.9) in the values from 6 to 100 ng/ml BDNF. **(B)** Trk kinase inhibitor K252a blocks the upregulation of ATF4 caused by BDNF in hippocampal neuron cultures. Cultures were pre-treated ± K252a for 1 h and then exposed to 25 ng/ml BDNF for 4 h in presence or absence of the inhibitor. Left panel shows Western immunoblot, right panel shows quantification relative to untreated control (means ± SEM) from the same blot. *p = 0.01 for control vs. BDNF, one-way ANOVA (*F* = 8.3) using the *post hoc* Tukey HSD test. The same test revealed *p* = 0.9 for control vs. K252a+BDNF. Comparable results were found in an independent experiment. **(C)** TrkB-Fc-IgG blocks the elevation of ATF4 protein promoted by BDNF. Cultured hippocampal neurons were pre-treated with control IgG or TrkB-Fc-IgG fusion protein for 1 h then treated with 25 ng/ml BDNF for 4 h and assessed by Western immunoblotting for ATF4 expression. Numbers below bands show relative optical density normalized to GAPDH loading control. Comparable results were achieved with cultured cortical neurons.

A dose-response study revealed a maximal effect of BDNF in the range of 6–100 ng/ml (about 30–500 pM BDNF dimer) in hippocampal neuron cultures (Figure 3A). This suggested the activation of “high affinity” TrkB receptors. To test this idea, we employed the Trk-kinase inhibitor K252a and found that this significantly blocked the effect of BDNF on ATF4 upregulation (Figure 3B) We additionally used TrkB-Fc-IgG1 to specifically sequester TrkB-binding proteins and this also repressed BDNF-induced elevation of ATF4 in hippocampal (Figure 3C) and cortical neurons (data not shown).

### BDNF Induces ATF4 mRNA, But Also Elevates ATF4 Protein by a Non-transcriptional Mechanism

**Figure 4:**
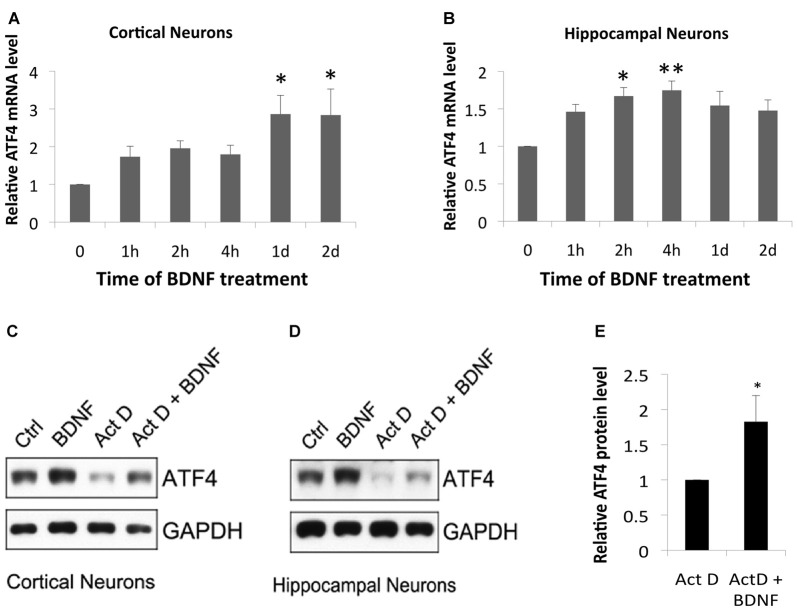
BDNF elevates ATF4 in neurons via both transcriptional and non-transcriptional mechanisms. **(A,B)** BDNF elevates *Atf4* mRNA in cultures of cortical **(A)** and hippocampal **(B)** neurons. Cultures (12 DIV) were exposed to 25 ng/ml BDNF for the indicated times and assessed by qPCR for *Atf4* mRNA levels (normalized to alpha-tubulin). Values represent means ± SEM for three (cortical cultures) or five (hippocampal cultures) independent experiments, with one lysate per time point and qPCR performed in triplicate. *p* values were calculated vs. no BDNF treatment using the Kruskal-Wallis test [overall *p* = 0.04 **(A)** and 0.037 **(B)**] with the *post hoc* Conover method adjusted by the FDR procedure of Benjamini-Hochberg. *p = 0.009 **(A)** and 0.031 **(B)**. **p = 0.014 **(B)**. **(C,D)** BDNF elevates ATF4 protein in cultured cortical and hippocampal neurons in part via a transcription-independent mechanism. Cultures were pre-treated ± the RNA synthesis inhibitor actinomycin-D (0.2 μM) for 0.5 h and then with or without 25 ng/ml BDNF ± actinomycin-D for 2 h and then total cell extracts were subjected to Western immunoblotting as indicated. **(E)** Quantification of relative ATF4 protein levels in hippocampal cultures treated with actinomycin-D alone or with actinomycin-D + BDNF indicates transcription-independent regulation of ATF4 expression. Values represent means ± SEM for four independent experiments, each with a single cell lysate per condition. *p = 0.004 (*t*-test).

We next used qPCR to determine whether BDNF regulates *Atf4* mRNA expression. BDNF significantly increased *Atf4* transcripts in cortical cultures at 1 and 2 days of treatment (Figure 4A) and in hippocampal cultures at 2 and 4 h (Figure 4B). The magnitudes of these effects on *Atf4* mRNA (a maximum of about 1.5–3-fold) were less than observed for ATF4 protein under similar conditions (Figure 1), raising the possibility that BDNF regulates ATF4 expression via both transcriptional and post-transcriptional mechanisms. To further assess whether there is a transcription-independent component of ATF4 regulation by BDNF, we treated cortical and hippocampal cultures with the transcription inhibitor actinomycin-D for 0.5 h and then with BDNF for 2 h in the continued presence of the drug. Although transcriptional blockade over the time course of the experiment substantially diminished basal ATF4 levels, BDNF was still able to elevate ATF4 protein levels under these conditions (Figures 4C–E). Quantification over four independent experiments with hippocampal neurons (Figure 4E) revealed a significant mean increase of about 1.8-fold.

### Elevation of ATF4 Protein by BDNF Does Not Involve Regulation of eIF2α Phosphorylation

**Figure 5:**
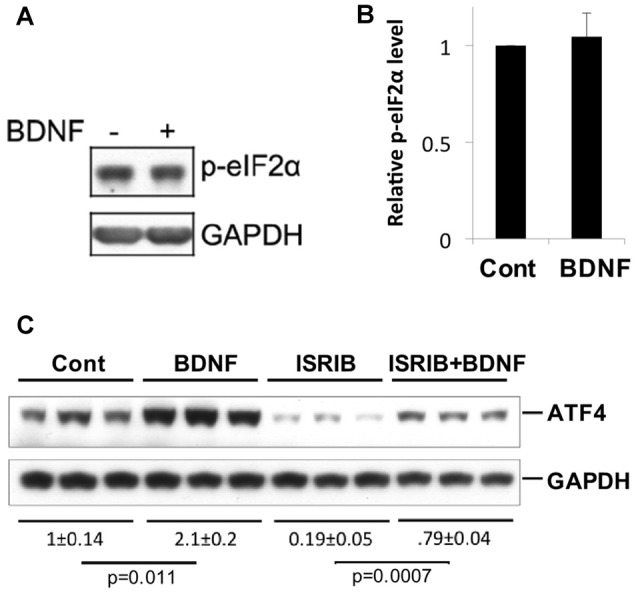
BDNF does not elevate ATF4 protein via a phospho-eIF2α-dependent mechanism. **(A,B)** BDNF treatment (4 h, 25 ng/ml) does not affect levels of phospho-eIF2α in cultures of hippocampal neurons. Left panel shows representative Western immunoblot, right panel quantification (mean ± SEM for five independent experiments, each performed on a single lysate per condition). **(C)** Inhibition of phospho-eIF2α-dependent protein synthesis by Integrated Stress Response inhibitor (ISRIB; 50 nM) does not block the capacity of BDNF to elevate ATF4 protein levels in cultured hippocampal neurons. Replicate cultures were pre-treated ± ISRIB or diluent (DMSO) for 1 h and then for an additional 4 h ± BDNF in the continued presence of diluent or ISRIB and then whole cell extracts were subjected to Western-immunoblotting. The blot image was scanned and relative levels of ATF4 (normalized to GAPDH as loading control) determined and indicated below the blot (values are means ± SEM for the three replicates for each condition). The indicated *p* values were calculated by the *t*-test. Similar results were achieved in two additional independent experiments.

A variety of cellular stresses increase ATF4 protein levels by stimulating phosphorylation of the eukaryotic translation initiation factor eIF2α (Bellato and Hajj, 2016; Pakos-Zebrucka et al., 2016). When phosphorylated under such conditions, eIF2α shows reduced capacity to support bulk protein synthesis, but greatly enhanced capacity to promote translation of *Atf4* and additional mRNAs with short up stream open reading frames in their 5′ UTR. Because elevation of eIF2α phosphorylation is a major mechanism by which ATF4 levels are rapidly increased in cells, we next asked whether BDNF affects the state of eIF2α phosphorylation in neurons under conditions in which it increases ATF4 protein. To achieve this, we treated hippocampal cultures with BDNF for 4 h, a time at which ATF4 induction is maximal, and assessed levels of phospho-eIF2α (Ser51; eIF2α-P) by western blotting. The data from five independent experiments showed no significant change in eIF2α phosphorylation in response to BDNF exposure (Figures 5A,B). To further assess the potential role of eIF2α phosphorylation in BDNF-promoted ATF4 regulation, we employed ISRIB (trans-N, N′-(Cyclohexane-1,4-diyl)bis(2-(4-chlorophenoxy)acetamide, trans-N,N′-1,4-Cyclohexanediylbis[2-(4-chlorophenoxy)-acetamide), a compound that blocks the effect of eIF2α phosphorylation on ATF4 translation by locking exchange factor eIF2B in an active conformation (Sidrauski et al., 2015a,b). Hippocampal cultures were pretreated with ISRIB for 1 h and then for an additional 4 h with the drug and BDNF. ISRIB alone substantially diminished (by about 80%) ATF4 protein expression over the course of the experiment (Figure 5C), indicating that eIF2α-P plays a major role in regulating basal ATF4 protein levels in cultured hippocampal neurons. Despite this, ISRIB did not block the capacity of BDNF to significantly elevate ATF4 protein expression (Figure 5C). Thus, BDNF appears to induce ATF4 protein levels by a mechanism that is independent of eIF2α phosphorylation.

### Transcriptome Analysis Reveals That Multiple ATF4 Targets Are Not Regulated by BDNF in an ATF4-Dependent Manner

Because ATF4 is a transcription factor, it might be anticipated that its up-regulation by BDNF would in turn result in changes in expression of ATF4-responsive genes. To assess this, we carried out RNA sequencing (RNAseq) analysis of transcripts in hippocampal neuron cultures that were infected with lentivirus expressing either a control or ATF4-directed shRNA for 5 days and then treated with BDNF for various times (0, 2, 4, 8, 16 and 24 h). The analysis confirmed that BDNF up-regulated *Atf4* mRNA by about 60% within 2–4 h and that shATF4 depleted *Atf4* transcripts by about 90% at all time points (data not shown).

In addition, we carried out gene chip transcriptome analysis of cortical cultures infected for 3 days with lentivirus expressing either shATF4 or ATF4 as well as with corresponding control viruses. In this study, there was an approximate 70% knockdown of ATF4 transcript levels by shATF4 and a doubling of ATF4 transcripts with ATF4 over-expression. The various experiments thus permitted us to identify genes in neurons regulated by ATF4 down-regulation or over-expression, genes regulated by BDNF, and genes regulated by BDNF in absence of ATF4 expression.

To assure that the presence of shATF4 did not globally disrupt normal BDNF signaling or gene responses, we first surveyed our RNAseq data for a number of early response genes that peaked by 2 h of BDNF treatment (*Arc, Egr2, 3, 4, Gadd45g, Jun, JunB, Klf10, Sertad1* and *Ier2*). These showed no significant differences in either basal or BDNF-induced (2 h) expression (Supplementary Table S1). We also detected a number of genes that underwent greater than 1.5-fold sustained induction by BDNF (up to 24 h) that also showed no significant effect of shATF4 on either basal or BDNF-stimulated (24 h) expression (*VGF, Cited1, Galnt7, EMP1, Sen1b, Col25a1, Gfra, Kcnab1, Kcnf1, Pde9a* and *Sh2d5*) (Supplementary Table S2).

To enhance the possibility of identifying direct ATF4 targets that might be significantly regulated by BDNF in an ATF4-dependent manner, we surveyed the top 124 BDNF-regulated genes previously described by ChIPseq to have binding peaks for ATF4 within 3 kb of the translation start site (Han et al., 2013). These included *Ddit3, Ier2, Sertad1, Jhdm1d, Tet3, Dusp6, Synj2, and Bhlhe22*. ATF4 knockdown did not significantly change the basal or BDNF-stimulated levels of these genes with the exceptions of *Dusp6 and Bhlhe22* in which cases the absolute basal and BDNF-stimulated values of expression were altered. However, in both cases, normalization to basal expression to take into account the effect of ATF4 knockdown indicated no influence of ATF4 on regulation by BDNF (Supplementary Figure S2).

We next assessed a number of genes (*Chac1, Eif4ebp1, Asns, Psph, Slc7a3, Slc7a5, Slc3a2, Atf5 and Phgdh*) that the literature has indicated as direct targets of ATF4 and that have been reported to be upregulated under stress conditions in an ATF4-dependent manner (Harding et al., 2003; Chen et al., 2004; Adams, 2007; Yamaguchi et al., 2008; Zhou et al., 2008; Mungrue et al., 2009; Han et al., 2013). As an indication that these genes are indeed ATF4-responsive in neurons, all underwent significant increases in expression in response to ATF4 over-expression (Supplementary Table S3) and in each case, RNAseq showed that the basal levels in neurons were diminished by shATF4 (Supplementary Figure S3). Despite the considerable elevation of ATF4 protein levels triggered by BDNF treatment, none of the above genes was substantially induced in response to BDNF in a manner that appeared to be dependent on ATF4 (Supplementary Figure S3). That is, in each case, although shATF4 decreased the basal level of mRNA expression, there was a similar-fold change in expression with BDNF irrespective of treatment with sh-control or shATF4.

We also considered expression of the tenascin-C (*Tnc*) and vascular endothelial growth factor A Vegfa (*Vegfa*) genes. It has been reported that the dimer of the PDGF B chain (PDGF-BB) and FGF2 upregulate ATF4 and that this in turn leads to ATF4-dependent induction of *Tnc* and *Vegfa* transcripts, respectively (Malabanan et al., 2008, 2012). In agreement with this, our RNAseq data indicated that shATF4 reduced Tnc and Vegfa transcript levels by about half in hippocampal neurons (Supplementary Figure S4). However, in the case of Tnc, we observed no major influence of BDNF either with or without ATF4 knockdown. For Vegfa, there was a maximal 60% elevation of transcripts with BDNF and the relative induction was somewhat enhanced rather than blocked by shATF4 (Supplementary Figure S4). Thus, although basal levels of these genes were influenced by ATF4, they were not induced by BDNF in an ATF4-dependent manner.

### Sestrin 2 (*Sesn2*) Undergoes ATF4-Dependent Regulation by BDNF

**Figure 6:**
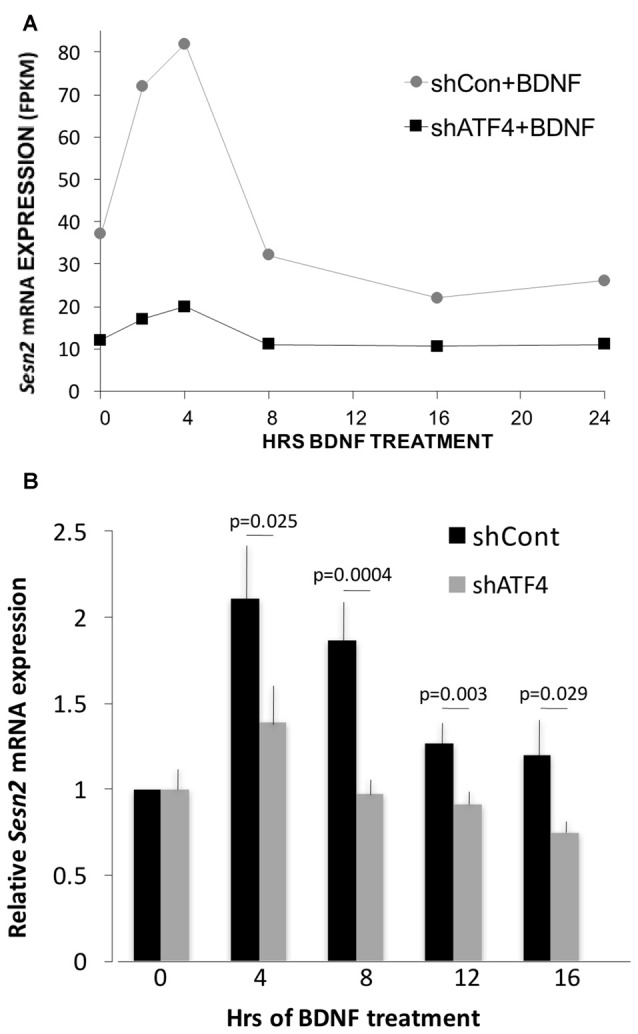
*Sesn2* undergoes ATF4-dependent regulation in response to BDNF treatment. **(A)** RNAseqderived fragments per kilobase per million (FPKM) values for *Sesn2* transcripts at various times of exposure to BDNF (25 ng/ml) with or without ATF4 knockdown. Hippocampal neurons were cultured for 7 days and then infected with lenti-shControl or lenti-shATF4. Five days after infection, cultures were treated with BDNF for the indicated times and harvested for RNA which was then subjected to RNAseq. **(B)** Cultures treated as above were subjected to qPCR for detection of *Sesn2* transcripts. Mean values for shControl and shATF4 at 0 time were set to 1 after normalization to alpha-tubulin (mean value for shATF4 was 0.81 ± 0.09 compared with shControl at time 0, *p* = 0.07 *t*-test, *n* = 8). Comparison of values for no BDNF treatment + shControl vs. BDNF + shControl (all time points) by the Kruskal-Wallis test yielded an overall *p* value of 0.001. The *post hoc* Conover method adjusted by the FDR procedure of Benjamini-Hochberg provided individual *p* values (vs. time 0) of 0.00006 (4 h and 8 h), 0.034 (12 h) and 0.09 (16 h). The same comparison for no BDNF treatment + shATF4 (all time points) yielded an overall *p* value of >0.05. The indicated *p* values on the figure are for each time point for shControl vs. shATF4 and were calculated by the *t*-test, *n* = 4–7 independent experiments, each performed with one sample per time point.

One ATF4 target gene that did show apparent ATF4-dependent regulation by BDNF was *Sesn2* (*Sestrin 2*). *Sesn2* is reported to inhibit both accumulation of reactive oxygen species and activation of mTorc1 (Kim et al., 2015) and to be a direct ATF4 target (Ye et al., 2015). It is involved in maintenance of metabolic homeostasis (Lee et al., 2012) as well as protection from death caused by stresses (Ben-Sahra et al., 2013; Chen et al., 2014; Saveljeva et al., 2016; Shi et al., 2017). As evidence that ATF4 regulates *Sesn2* in hippocampal neurons, RNAseq indicated that *Sesn2* transcript levels were reduced in cultures after ATF4 knockdown (Figure 6A). In accord with a recent report (Wu et al., 2016), RNAseq also showed that *Sesn2* responded to BDNF, with a doubling of mRNA levels by 2–4 h (Figure 6A). The RNAseq data also indicated that the response of *Sesn2* was blunted in cells in which ATF4 was knocked down (Figure 6A). To verify and extend these results, we carried out additional independent experiments in which qPCR was used to quantify relative *Sesn2* transcript levels in BDNF-treated cultures infected with lentivirus expressing shControl or shATF4. These experiments corroborated that BDNF upregulates *Sesn2* transcript levels and that the response is significantly repressed by ATF4 knockdown (Figure 6B).

## Discussion

### BDNF Regulates Both ATF4 mRNA and Protein Expression in Neurons

In the present study we report that BDNF, via activation of TrkB receptors, promotes a rapid and sustained elevation of the transcription factor ATF4 in both cortical and hippocampal neurons and does so at the levels of both transcripts and protein. Our findings further indicate that BDNF induces elevated expression of *Sesn2* and that this is mediated by ATF4.

Much of the effort in understanding ATF4 regulation has focused on its translational control by eIF2α (Bellato and Hajj, 2016). The present findings with ISRIB as well as past studies indicate that basal ATF4 levels in neurons and brain are controlled at least in part by an eIF2α-P-dependent pathway (Costa-Mattioli et al., 2007; Trinh et al., 2012; Bellato and Hajj, 2016). In contrast, our findings appear to rule out elevation of eIF2α phosphorylation as a mechanism by which BDNF elevates ATF4 levels. We did not detect BDNF-dependent changes in eIF2α phosphorylation, and induction of ATF4 protein by BDNF was not suppressed by ISRIB, an agent that inhibits the effects of eIF2α phosphorylation on selective translation of mRNAs, including *Atf4* (Sidrauski et al., 2015a,b). Consistent with this, at least part of the effect of BDNF on ATF4 levels appeared to be due to elevation of *Atf4* transcripts. However, the increase in mRNA levels appeared to be less robust than for protein, suggesting additional mechanisms of regulation. In this regard, immunostaining revealed rapid elevation of ATF4 in processes as well as in cell bodies. The presence of substantial ATF4 levels in processes and its rapid elevation there by BDNF suggest the possibility of elevated local translation as has been seen in the case of Abeta 1–42-promoted ATF4 synthesis in axons (Baleriola et al., 2014). There are several prior reports of instances in which ATF4 protein is induced independently of eIF2α-P, but the mechanisms by which this occurs have yet to be defined (Lehman et al., 2015; Ishizawa et al., 2016).

There are few studies showing that growth factors regulate ATF4 levels. It was reported that FGF-2 elevated ATF4 mRNA and protein in vascular smooth muscle cells by about 2.5-fold and increased ATF4 occupancy of an ATF4 recognition element in the *VEGFA* gene promoter (Malabanan et al., 2008). ATF4 knockdown also blocked FGF-2 induction of VEGFA mRNA. It was further demonstrated that PDGF-BB induces similar upregulation of ATF4 mRNA and protein and that ATF4 knockdown inhibited PDGF-BB-promoted induction of *Tnc* mRNA (Malabanan et al., 2012). Our findings now show that BDNF, a neurotrophin family member, can also modulate ATF4 levels and can do so in post-mitotic neurons. However, the effects on downstream targets appear to be cell-type and/or growth factor specific. Thus, while basal *Tnc* and *Vegfa* transcript levels were diminished in cortical neurons by shATF4, BDNF did not regulate them in an ATF4-dependent manner.

### *Sesn2*, But Not Many Other ATF4 Targets, Undergoes ATF4-Dependent Regulation by BDNF

A somewhat unanticipated and surprising outcome of our studies was that despite a marked increase in ATF4 levels in BDNF-treated neurons, we did not detect ATF4-dependent changes in expression of a number of genes that have been identified as ATF4 targets. This did not appear due to failure of ATF4 to reach the nucleus because our immunostaining studies detected BDNF-dependent increases in ATF4 there and because at least one gene (*Sesn2*) showed ATF4-dependent regulation by BDNF. Potential reasons might include limited expression of essential co-regulatory proteins, the presence or absence of post-transcriptional ATF4 modifications that alter its transcriptional activity, and BDNF-promoted mechanisms that actively suppress induction of most ATF4-responsive genes. Irrespective of the underlying mechanisms, it is evident that elevation of ATF4 levels caused by BDNF or by stress do not necessarily have the same consequences.

We identified *Sesn2* as a BDNF-responsive gene whose induction was greatly diminished by ATF4 knockdown. Prior work has identified *Sesn2* as an ATF4-regulated gene in the context of cellular stresses (Lee et al., 2012; Ben-Sahra et al., 2013; Chen et al., 2014; Ye et al., 2015; Garaeva et al., 2016; Saveljeva et al., 2016; Wu et al., 2016; Shi et al., 2017) and putative ATF4 binding sites have been found in the *Sesn2* promoter (Han et al., 2013; Ding et al., 2016; Kersey et al., 2016).

A recent study also reported elevation of *Sesn2* in cultured cortical neurons in response to BDNF (Wu et al., 2016). In this case, the authors described a pathway in which *Sesn2* induction by BDNF included NFκB promoted transcription. The authors also noted that NFκB signaling did not appear to account for the entire effect of BDNF on *Sesn2* and thus could not rule out the involvement of additional transcription factors. Our findings indicate that ATF4 significantly contributes to *Sesn2* induction by BDNF and appears to account for the bulk of this effect under our experimental conditions. It remains to be seen whether or not ATF4 and NFκB play independent roles in mediating the response of *Sesn2* to BDNF.

Sestrin2 is reported to function as an anti-oxidant protein by reducing accumulation of reactive oxygen species and as a suppressor of mTorc1 kinase (Lee et al., 2012; Ben-Sahra et al., 2013; Chen et al., 2014; Ye et al., 2015; Garaeva et al., 2016; Saveljeva et al., 2016; Wu et al., 2016; Shi et al., 2017). These activities appear to emanate from separate functional domains (Kim et al., 2015) and place Sestrin2 as an important regulator of cellular metabolic and redox homeostasis. Several prior studies have indicated roles for Sestrin2 in neurons by protecting them from various types of stresses dues to its anti-oxidant and mTorc1 inhibitory actions (Chen et al., 2014; Wang et al., 2015; Shi et al., 2017). In this context, stimulation of the ATF4-*Sesn2* pathway by BDNF could play a role in the latter’s known neuroprotective actions. In response to a variety of stresses, ATF4 activates both protective and pro-apoptotic genes. The capacity of BDNF to activate ATF4-*Sesn2* without apparent induction of pro-apoptotic ATF4 targets such as *Ddit3*/CHOP, *Sertad1* and *Chac1* suggests an alternative pathway for neural protection without risk of triggering neural degeneration.

We have reported that experimental depletion of ATF4 from neurons leads to deficits in memory as well as to impairments in dendritic spine density, synapse formation, LTP and LTD (Liu et al., 2014; Pasini et al., 2015). In this context, BDNF may play an important role in “safely” maintaining ATF4 at levels required for optimal brain function without triggering its pro-apoptotic actions.

## Author Contributions

JL designed, performed and analyzed the experiments shown in Figures 1, 3, 4, 6 and Supplementary Figure S2 and in Supplementary Tables S1–S3. FA designed, performed and analyzed the experiments shown in Figure 2 and Supplementary Figure S1. RWLS designed, performed and analyzed the experiments shown in Figure 5. CC prepared hippocampal and cortical neuron cultures and prepared lentiviral shRNA. PLN participated in design of experiments involving RNAseq and coordinated RNA sequencing and analysis of RNAseq data. SJA performed analysis of RNAseq data. MLS and LAG conceived and coordinated the study and drafted the article. All authors reviewed the manuscript, contributed to revising it for important intellectual content, gave final approval for publication and agree to be accountable for all aspects of the work in ensuring that questions relating to the accuracy or integrity of any part of the work are appropriately investigated and resolved.

## Conflict of Interest Statement

The authors declare that the research was conducted in the absence of any commercial or financial relationships that could be construed as a potential conflict of interest.

## Footnotes

^1^http://bioinfo.lifl.fr/RNA/sortmerna/

^2^http://www.bioinformatics.babraham.ac.uk/projects/trim_galore/

^3^http://astatsa.com/KruskalWallisTest/

^4^http://astatsa.com/OneWay_Anova_with_TukeyHSD/
