# Supplementary material for: Brain-Derived Neurotrophic Factor Elevates Activating Transcription Factor 4 (ATF4) in Neurons and Promotes ATF4-Dependent Induction of Sesn2
Source: Front Mol Neurosci. 2018 Mar 1;11:62. doi: 10.3389/fnmol.2018.00062 (PMC5863619; doi:10.3389/fnmol.2018.00062)
Supplement: Supplementary file 1 [file Data_Sheet_1.DOCX]

*Supplementary Material*

Brain-Derived Neurotrophic Factor Elevates Activating Transcription Factor 4 (ATF4) in Neurons and Promotes ATF4-Dependent Induction of *Sesn2*

**Jin Liu, Fatou Amar, Carlo Corona, Raphaella W. L. So, Stuart J. Andrews, Peter L. Nagy, Michael L. Shelanski, and Lloyd A. Greene***

***Correspondence:** Corresponding Author: lag3@cumc.columbia.edu

**Supplementary Figures and Tables**


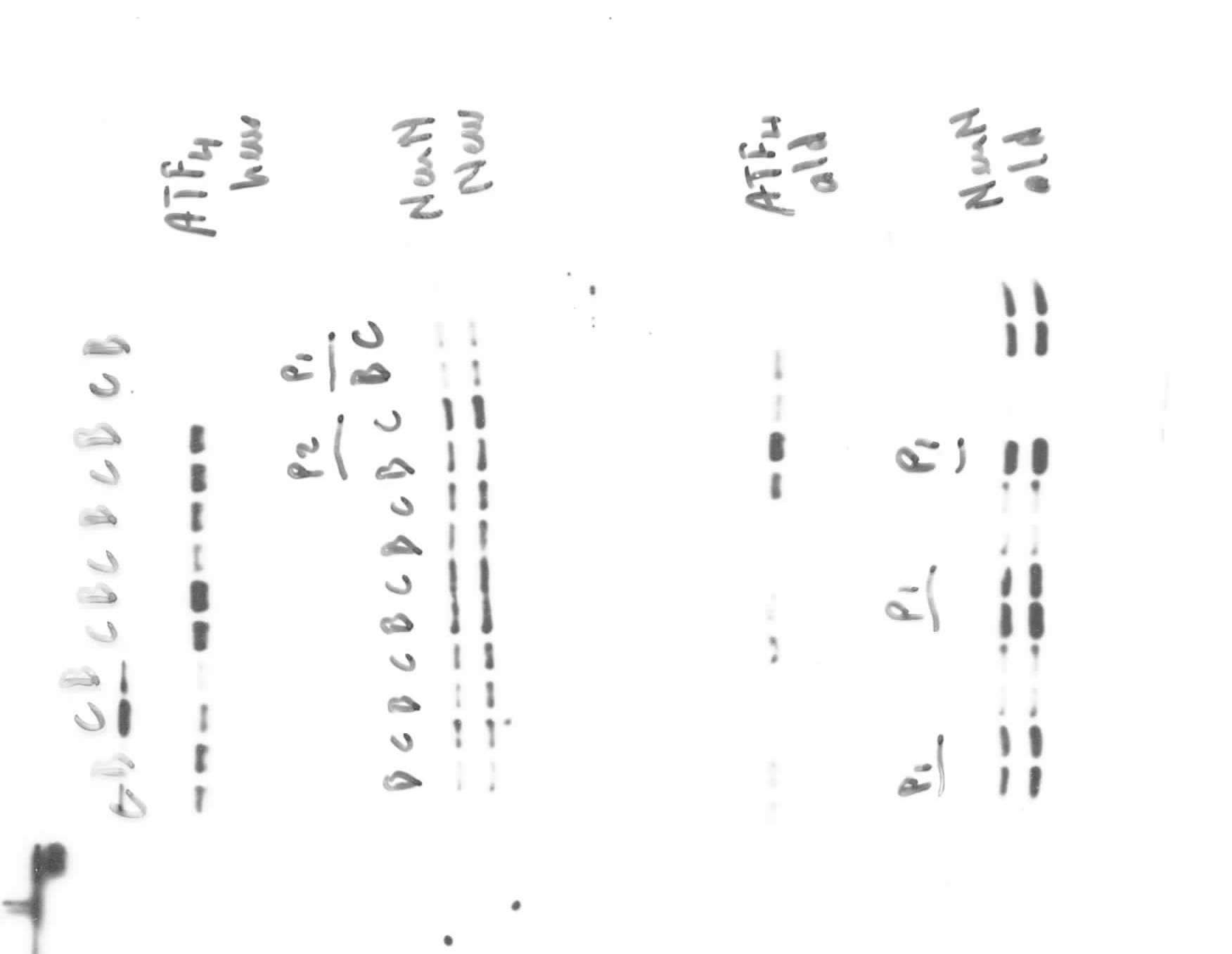


**BDNF - + - + - + - +**

**NeuN**

P1

P2

P1

P2

**Supplementary Figure 1**. Enrichment of NeuN protein in the nuclear-enriched (P1) fraction of hippocampal neurons used for measurement of ATF4 protein values. Details of the experiment are as given in the legend of Figure 2.


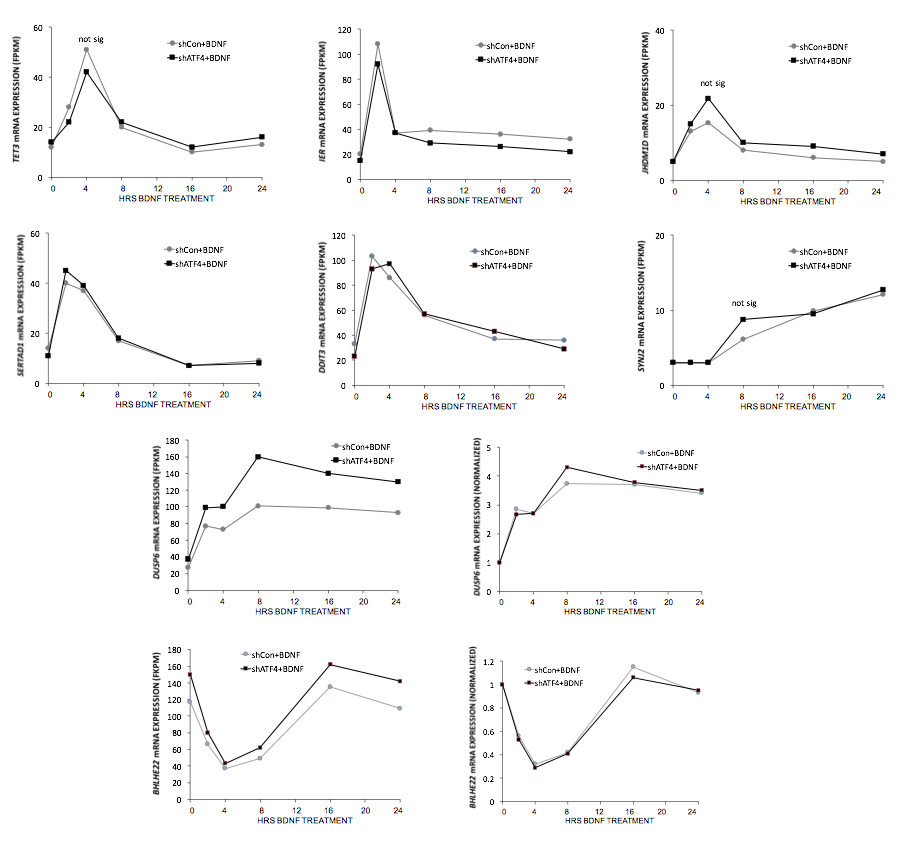


**Supplementary Figure 2**. BDNF up-regulates multiple genes reported to be ATF4 targets, but in an ATF4-independent manner. RNA-seq was carried out on shControl and shATF4-treated hippocampal neuron cultures (3 replicate cultures) after various times of BDNF exposure as described in the text. Values are given as FPKM and, in the cases of *Dusp6 and Bhlhe22,* are also shown normalized to correct for the differences in FPKM values at time 0.


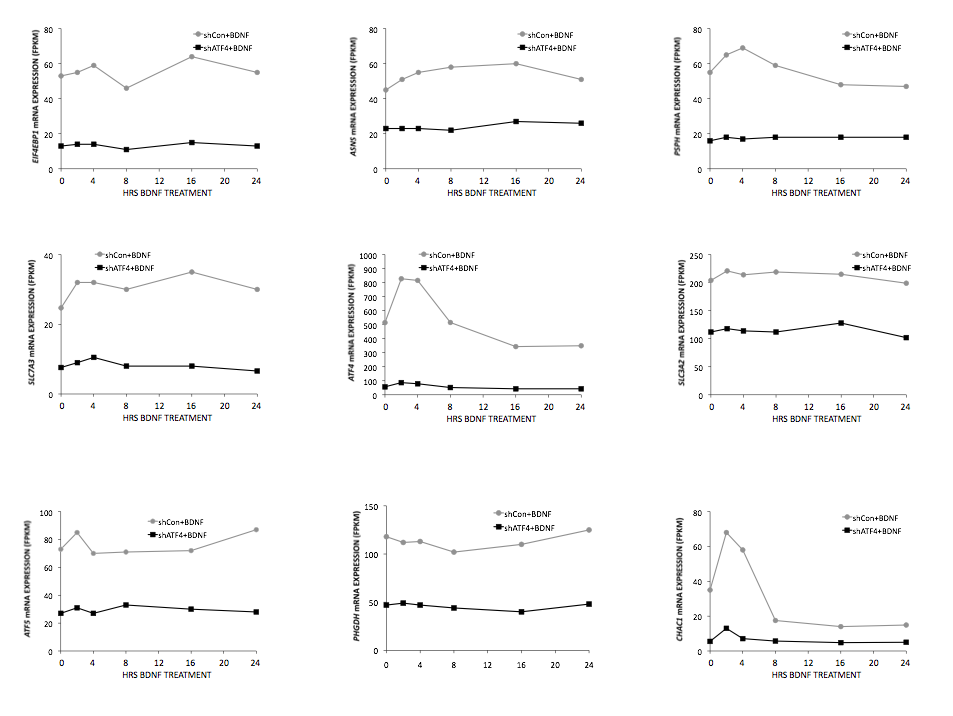


**Supplementary Figure 3**. ATF4 knockdown identifies genes in cultured hippocampal neurons whose basal expression is ATF4-dependent, but that do not undergo substantial ATF4-dependent expression changes in response to BDNF. RNA-seq was carried out on shControl and shATF4-treated cortical hippocampal cultures in triplicate after various times of BDNF exposure as described in the text.


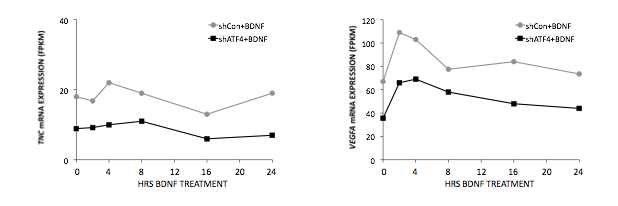


**Supplementary Figure 4**. ATF4 knockdown diminishes basal expression of *Tnc* and *Vegfa* in cultured hippocampal neurons, but these transcripts do not undergo ATF4-dependent regulation by BDNF. RNAseq was carried out on shControl and shATF4-treated hippocampal neuron cultures iin triplicate after various times of BDNF exposure as described in the text.

**Supplementary Table 1**: ATF4 knockdown does not significantly affect regulation of genes maximally induced by BDNF at 2 h in cultured hippocampal neurons. Data from RNAseq analysis carried out on shControl and shATF4-treated hippocampal neuron cultures in triplicate after 2 h of BDNF exposure as described in the text.

| Gene | FPKMshCont | FPKMshATF4 | p |  | FPKMshCont  +2h BDNF | FPKMshATF4  + 2h BDNF | p |
| --- | --- | --- | --- | --- | --- | --- | --- |
|  |  |  |  |  |  |  |  |
| Arc | 12.5 | 9.7 | .37 |  | 328 | 300 | .46 |
| Egr2 | 3.6 | 4.1 | .16 |  | 75 | 71 | .64 |
| Egr3 | 14 | 17 | .29 |  | 163 | 166 | .87 |
| Egr4 | 10 | 10 | .77 |  | 105 | 86 | .14 |
| Gadd45g | 32 | 25 | .14 |  | 98 | 90 | .59 |
| Jun | 37 | 30 | .18 |  | 123 | 122 | .98 |
| JunB | 59 | 51 | .33 |  | 283 | 306 | .54 |
| Klf10 | 17 | 17 | .77 |  | 78 | 99 | .07 |
| Sertad1 | 14 | 11 | .17 |  | 40 | 45 | .36 |
| Ier2 | 20 | 15 | .2 |  | 109 | 92 | .27 |

**Supplementary Table 2**: ATF4 knockdown does not significantly affect regulation of genes maximally induced by BDNF at 24 h in cultures of hippocampal neurons. Data from RNA-seq analysis carried out on shControl and shATF4-treated hippocampal neuron cultures in triplicate after 24 h of BDNF exposure as described in the text.

| Gene | FPKMshCont | FPKMshATF4 | p |  | FPKMshCont  +24h BDNF | FPKMshATF4  + 24h BDNF | p |
| --- | --- | --- | --- | --- | --- | --- | --- |
|  |  |  |  |  |  |  |  |
| Vgf | 60 | 66 | .51 |  | 180 | 187 | .78 |
| Cited1 | 5.8 | 4.0 | .16 |  | 26 | 26 | .91 |
| Galnt7 | 8.7 | 6.1 | .06 |  | 15 | 17 | .58 |
| Emp1 | 2.2 | 1.4 | .07 |  | 18 | 21 | .39 |
| Scn1b | 25 | 32 | .12 |  | 56 | 59 | .82 |
| Col25a1 | 6.8 | 7.3 | .73 |  | 15 | 22 | .06 |
| Gfra1 | 10.7 | 9.4 | .47 |  | 23 | 24 | .81 |
| Kcnab1 | 16 | 13 | .19 |  | 38 | 31 | .17 |
| Kcnf1 | 14 | 12 | .52 |  | 55 | 58 | .73 |
| Pde9a | 5.6 | 7.4 | .18 |  | 12 | 15 | .26 |
| Sh2d5 | 7.6 | 7.4 | .91 |  | 12 | 13 | .76 |

**Supplementary Table 3**. ATF4 over-expression (O.E.) up-regulates known ATF4 targets in cultured cortical neurons. Data are from Gene chip analysis carried out on triplicate cultures as described in the text. Values are means ± SEM (n=3) for expression relative to that in cultures infected with shControl.

| Gene | Fold increase  (ATF4 O.E.) |
| --- | --- |
|  |  |
| Chac1 | 8.9±2 |
| Eif4ebp1 | 6.1±0.4 |
| Asns | 5.4±0.6 |
| Psph | 4.1±0.2 |
| Slca3 | 6.5±0.4 |
| Slc7a5 | 3.7±0.6 |
| Slc3a2 | 3.0±0.04 |
| Atf5 | 3.8±0.2 |
| Phgdh | 4.3±1.4 |
